# Factors associated with blood loss in ankylosing spondylitis patients with hip involvement undergoing primary total hip arthroplasty: a cross-sectional retrospective study of 243 patients

**DOI:** 10.1186/s13018-020-02064-z

**Published:** 2020-11-18

**Authors:** Liangliang Li, Jun Fu, Chi Xu, Haitao Guan, Ming Ni, Wei Chai, Libo Hao, Yonggang Zhou, Jiying Chen

**Affiliations:** 1 Medical School of Chinese PLA, Beijing, China; 2 Department of Orthopaedics, The First Medical Centre, Chinese PLA General Hospital, Beijing, China; 3 Department of Orthopaedics, The Second Hospital of Shanxi Medical University, Taiyuan, Shanxi China

**Keywords:** Ankylosing spondylitis, Hip involvement, Total hip arthroplasty, Blood loss

## Abstract

**Background:**

Total hip arthroplasty (THA) can cause considerable blood loss and perioperative transfusion in ankylosing spondylitis (AS) patients. This study aimed to identify the factors related to blood loss in AS patients with hip involvement undergoing THA.

**Methods:**

We analyzed 243 AS patients with advanced hip involvement undergoing primary THA from 2012 to 2017. Bilateral THA was performed by a one-stage operation during one general anesthesia session. The patients were divided into three groups according to the grade of blood loss, as determined by the Advanced Trauma Life Support hypovolemic shock classification system. Ordinal logistic regression was used to identify factors associated with blood loss in the patients.

**Results:**

The proportion of patients who were male, underwent bilateral THA, had a hip range of motion (ROM) = 0°, had a BASRI-hip score of 4, underwent iliopsoas and adductor release, and underwent autologous or allogenic transfusion increased significantly with the grade of blood loss, while that of the patients who received tranexamic acid (TXA) decreased significantly (*P* < 0.05). The preoperative hemoglobin (Hb) level, hematocrit level, and operating time also increased significantly with the grade of blood loss (*P* < 0.05). The ordinal logistic regression results identified the factors related to blood loss during THA in AS patients with hip involvement to be the male sex (odds ratio [OR] = 3.287; 95% confidence interval [CI] 1.022, 10.567), bilateral THA (OR = 13.896; 95% CI 4.950, 39.011), hip ROM = 0° (OR = 2.513; 95% CI 1.277, 4.946), an elevated erythrocyte sedimentation rate (ESR) level (OR = 3.042; 95% CI 1.320, 7.014), an elevated preoperative Hb level (OR = 1.043; 95% CI 1.017, 1.070), a long operating time (OR = 1.009; 95% CI 1.003, 1.016), and the administration of TXA (OR = 0.252; 95% CI 0.134, 0.472).

**Conclusions:**

The male sex, bilateral THA, a hip ROM = 0°, an elevated ESR level, a high preoperative Hb level, and a long operating time are risk factors associated with blood loss in AS patients with hip involvement undergoing THA, while the administration of TXA is a protective factor. These results might help determine the risk of bleeding in the perioperative assessment and develop more efficient blood management strategies for THA in AS patients with hip involvement.

## Background

Ankylosing spondylitis (AS) is an autoimmune disease that mainly affects the axial skeleton and can affect peripheral joints [1, 2]. The hip is the peripheral joint that is commonly involved, and hip involvement often results in severe deformities and functional disabilities due to stiffness or ankylosis [1, 2]. For advanced hip involvement, total hip arthroplasty (THA) has been proven to be an effective treatment [3–7]. Although there have been continuous improvements in anesthetic and surgical techniques, the procedure can cause considerable blood loss and perioperative transfusion. Until now, being a male, having experienced general anesthesia, having an American Society of Anesthesiologists (ASA) score of 3, having a Charlson comorbidity index > 3, having donated autologous blood preoperatively [8, 9], and having an international normalized ratio (INR) ≥ 1.25 [10] have been identified as clinical predictors for blood loss during THA.

In addition to these general factors, for AS patients, bleeding during THA is closely related to some characteristics of the disease itself, which mainly include the effect of systemic inflammatory activity on microvascular function and the coagulation system [11, 12] and the complexity of hip diseases [13]. Conventionally, the perioperative use of hemostatic [14, 15] and anticoagulant drugs [16] is also taken into account. Several studies have shown that bony ankylosis in the hip [13] and a higher disease activity [17] can cause an increase in bleeding during THA in AS patients, while a lower body mass index (BMI) is responsible for greater hidden blood loss postoperatively and a higher transfusion rate [18]. However, the comprehensive effects and interrelationships among these characteristic factors related to blood loss during THA in AS patients have not been reported, and whether any other factors may exist remains unclear. Thus, the current study was designed to identify the factors associated with blood loss in AS patients with hip involvement undergoing THA.

## Methods

### Participants and data sources

We retrospectively analyzed the data of AS patients with hip involvement who underwent primary THA in our orthopedic department from January 2012 to December 2017. This study was approved by the ethics committee of our hospital, and all the data were obtained from medical records and radiographs. AS was diagnosed on the basis of the 1984 modified New York criteria [19]. Patients who had coagulation disorders, thrombocytopenia, platelet dysfunction, a preoperative hemoglobin (Hb) level < 90 g/L, or other hematological diseases; patients who were administered erythropoietin; and patients with chronic diseases affecting hemodynamics, such as arrhythmia and hypertension, were excluded from the study. Patients with a proximal femur deformity or a history of hip surgery or non-primary THA were excluded as well. Ultimately, a total of 243 AS patients were enrolled. The severity of hip involvement was evaluated by the Bath Ankylosing Spondylitis Radiology Hip Index (BASRI-hip) [20] by two trained readers, according to preoperative anteroposterior pelvic radiographs. A BASRI-hip score ≥ 3 was considered the radiographic indication for THA.

### Surgical technique and perioperative treatment

The surgical indications included advanced hip involvement with pain and/or a case of hip ankylosis that limited one’s ability to perform daily activities and did not respond to nonsurgical treatment. Nonsteroidal anti-inflammatory drugs were discontinued at least 2 weeks before surgery. General anesthesia, the posterolateral approach, and a cementless hip prosthesis were used for all the patients. The ASA class was determined by anesthesiologists. At the start of the study period, we were not using tranexamic acid (TXA) routinely, but during the course of the study period, our practice changed and we now give an intravenous injection of 1 g 5–10 min before the skin incision [14, 15]. TXA was used in 65.4% of the patients in this study. A single drain was used at one operation site and was not used for more than 48 h postoperatively. Low-molecular-weight heparin or rivaroxaban was used for thromboprophylaxis at 10~12 h postoperatively, and rivaroxaban was taken orally after discharge for 35 days after the operation. For patients with a BMI < 18.5 kg/m^2^ or APTT exceeding the top of the normal range, only mechanical anticoagulation treatment was performed.

### Calculation of blood loss (CBL) and transfusion management

Total blood loss was calculated by the following formula, as reported by Mercuriali et al. [21], where RBC and Hct are the abbreviations for red blood cell and hematocrit, respectively:

$$ \mathrm{CBL}\ \left(\mathrm{mL}\ \mathrm{of}\ \mathrm{RBCs}\right)=\mathrm{mL}\ \mathrm{of}\ \mathrm{blood}\ \mathrm{volume}\ \left(\mathrm{BV}\right)\times \left({\mathrm{Hct}}_{\mathrm{preoperative}}-{\mathrm{Hct}}_{\mathrm{day}\ 5\ \mathrm{postoperative}}\right)+\mathrm{mL}\ \mathrm{of}\ \mathrm{RBC}\ \mathrm{transfused} $$

The RBC transfused volume was calculated as the sum of the autologous and allogenic transfusions. The predicted BV was calculated by the following formula, as described by Nadler et al. [22]:

$$ \mathrm{BV}\ \left(\mathrm{mL}\right)=\left(\mathrm{k}1\times {\mathrm{height}}^3+\mathrm{k}2\times \mathrm{weight}+\mathrm{k}3\right)\times 1000, $$

where height is measured in meters and weight is measured in kilograms. For men, k1 = 0.3669, k2 = 0.03219, and k3 = 0.6041; for women, k1 = 0.3561, k2 = 0.03308, and k3 = 0.1833.

Patients with a preoperative Hb level of at least 150 g/L or 130~150 g/L aged less than 65 years were approved for an autologous blood donation [23]. The intraoperative blood loss was monitored, and the transfusion was managed by an anesthesiologist, and the postoperative criteria for a transfusion were a Hb level < 70 g/L or the presence of anemic manifestations, such as a decrease in blood pressure (< 90/60 mmHg), pale lips, dizziness, weakness, and shortness of breath.

### Grouping criteria and assessment indicator

The patients were divided into three groups by the grade of blood loss (grade I~III < 750 ml, 750~1500 ml, and > 1500 ml), as determined by the Advanced Trauma Life Support (ATLS) hypovolemic shock classification system [24]. Clinical characteristics, preoperative laboratory values, and perioperative data were compared among the three groups. The clinical characteristics included age, sex, BMI, disease duration, bilateral THA, hip range of motion (ROM) = 0°, and a BASRI-hip of 4 (on any one side). The preoperative laboratory values included the erythrocyte sedimentation rate (ESR), C-reactive protein (CRP) level, thromboplastin time (TT), activated partial thromboplastin time (APTT), prothrombin time (PT), prothrombin activity (PT%), INR, fibrinogen concentration, platelet level, Hb level, and hematocrit (Hct). Elevated ESR and CRP levels were defined as follows: ESR > 15 mm/h for males and > 20 mm/h for females and CRP > 1 mg/dL. The perioperative data comprised data on iliopsoas and adductor release, the administration of TXA, chemoprophylaxis for thrombosis, the operating time, autologous transfusion, allogenic transfusion, and the ASA class.

### Statistical analysis

One-way ANOVA was used to compare continuous, normally distributed variables (mean ± standard deviation), while the Wilcoxon rank sum test was used to compare non-normally distributed, continuous variables (median, minimum to maximum). The chi-square test or Fisher’s exact test was used to compare the categorical and dichotomous variables. Ordinal logistic regression was performed to identify factors associated with blood loss in AS patients with hip involvement undergoing THA. All statistical analyses were performed with IBM SPSS statistics for Windows, version 25.0 (IBM, Armonk, NY, USA). A *P* value < 0.05 was considered statistically significant.

## Results

The admission age, disease duration, and BMI ranged from 19 to 76 years (median, 32 years), 0.5 to 40.3 years (median, 11 years), and 11.87 to 37.11 kg/m^2^ (median, 22.64 kg/m^2^), respectively. There were 218 (89.7%) male patients. A total of 163 (67.1%) patients underwent bilateral THA, 121 (49.8%) patients had a ROM = 0° in the hip, and 38 (15.6%) patients had a history of smoking. Of all the involved hips, there were 178 (43.8%) and 228 (56.2%) hips with BASRI-hip scores of 3 and 4, respectively. Iliopsoas and adductor release were performed in 41 (16.9%) patients, periprosthetic fracture occurred in 10 (4.1%) patients, TXA was administered in 159 (65.4%) patients, and chemoprophylaxis for thrombosis was administered in 183 (75.3%) patients. Autologous and allogenic transfusions were performed in 66 (27.2%) and 229 (94.2%) patients, 13.8% and 32.5% and 83.8% and 99.4% of whom underwent unilateral and bilateral THA, respectively. The operating time (90, 55 to 285 min vs 205, 70 to 455 min) and blood loss (790 ± 342 mL vs 1435 ± 495 mL) were also calculated for the patients who underwent unilateral and bilateral THA separately (Table 1).

**Table 1:** Baseline characteristics of the AS patients with hip involvement undergoing THA

Baseline characteristic	Values^a^	Range^b^
Clinical characteristics		
Age (years)	32	19 to 67
Male (n, %)	218 (89.7%)	
BMI (kg/m^2^)	22.64	11.87 to 37.11
Disease duration (years)	11	0.5 to 40.3
Hospital stay (days)	11	6 to 46
Smoking history (*n*, %)	38 (15.6%)	
Bilateral THA (*n*, %)	163 (67.1%)	
Hip ROM = 0°(*n*, %)	121 (49.8%)	
BASRI-hip (number of cases at involved hip)		
3 (*n*, %)	178 (43.8%)	
4 (*n*, %)	228 (56.2%)	
Preoperative laboratory values		
Elevated ESR level (> 15 mm/h for male and > 20 mm/h for female, *n*, %)	132 (54.3%)	1 to 99
Elevated CRP level (> 1 mg/dL, *n*, %)	165 (67.9%)	0.1 to 11.6
TT (s)	16.1 ± 1.0	12.3 to 19.4
APTT (s)	40.4 ± 4.9	30.3 to 54.0
PT (s)	13.5 ± 0.8	11.4 to 16.2
PT (%)	94.4 ± 12.0	63.6 to 123.0
INR	1.05 ± 0.08	0.88 to 1.30
Fibrinogen level (g/L)	4.23	1.76 to 13.10
Platelet level (× 10^9^/L)	273 ± 72	103 to 509
Hb level (g/L)	133 ± 16	90 to 171
Hct (L/L)	0.404 ± 0.043	0.284 to 0.512
Perioperative data		
Periprosthetic fracture (*n*, %)	10 (4.1%)	
Iliopsoas and adductor release (*n*, %)	41 (16.9%)	
Administration of TXA (*n*, %)	159 (65.4%)	
Chemoprophylaxis of thrombosis (*n*, %)	183 (75.3%)	
Autologous transfusion (*n*, %)	66 (27.2%)	
Unilateral THA (*n* = 80)	11 (13.8%)	
Bilateral THA (*n* = 163)	53 (32.5%)	
Allogenic transfusion (*n*, %)	229 (94.2%)	
Unilateral THA (*n* = 80)	67 (83.8%)	
Bilateral THA (*n* = 163)	162 (99.4%)	
Operating time (min)	180	55 to 455
Unilateral THA	90	55 to 285
Bilateral THA	205	70 to 455
CBL (mL)	1223 ± 542	162 to 3098
Unilateral THA	790 ± 342	162 to 1901
Bilateral THA	1435 ± 495	453 to 3098

*AS* ankylosing spondylitis, *THA* total hip arthroplasty, *BMI* body mass index, *ROM* range of motion, *BASRI-hip* Bath Ankylosing Spondylitis Radiology Hip Index, *ESR* erythrocyte sedimentation rate, *CRP* C-reactive protein, *TT* thromboplastin time, *APTT* activated partial thromboplastin time, *PT* prothrombin time, *PT%* prothrombin activity, *INR* international normalized ratio, *Hb* hemoglobin, *Hct* hematocrit, *TXA* tranexamic acid, *BV* blood volume, *CBL* calculated blood loss

^a^Mean ± SD or median

^b^Minimum to maximum

The proportion of patients who were male, underwent bilateral THA, had a hip ROM = 0°, had a BASRI-hip score of 4, underwent iliopsoas and adductor release, underwent autologous transfusion, and underwent allogenic transfusion increased significantly with the grade of blood loss, while that of patients who were administered tranexamic acid (TXA) decreased significantly (*P* < 0.05). There were significant differences in TT, APTT, fibrinogen level, platelet level, and proportion of patients who underwent chemoprophylaxis for thrombosis among the three groups (*P* < 0.05). The frequency of an elevated ESR in group II was higher than those in the other two groups, and the difference approached statistical significance (*P* = 0.058). The same trend was found in the frequency of an elevated level of CRP, but the difference was not significant (*P* = 0.168). The Hb level, Hct level, and operating time also increased significantly with the grade of blood loss (*P* < 0.05). No significant differences were found in BMI, disease duration, PT, PT %, INR, or ASA class among the three groups (*P* > 0.05) (Table 2).

**Table 2:** Comparison of the clinical characteristics, preoperative laboratory values, and perioperative data among the 3 groups stratified by the grade of blood loss, as determined by the ATLS hypovolemic shock classification system

	Grade of blood loss			*P* value
	I < 750 ml (*n* = 49)	II 750~1500 ml (*n* = 121)	III> 1500 ml (*n* = 73)	
Clinical characteristics				
Age (years)	32 (19 to 67)	32 (19 to 59)	34 (22 to 53)	0.449 ^c^
Male (*n*, %)	37 (75.5%)	110 (90.9%)	71 (97.3%)	< 0.0001^a^
BMI (kg/m^2^)	21.83 (11.87 to 36.73)	22.76(15.06 to 36.98)	23.39 (15.22 to 37.11)	0.078 ^c^
Disease duration (years)	10 (0.5 to 40.3)	10.5 (1.0 to 30.0)	14 (0.5 to 34)	0.114 ^c^
Bilateral THA (*n*, %)	8 (16.3%)	84 (69.4%)	71 (97.3%)	< 0.0001^a^
Hip ROM = 0° (*n*, %)	10 (20.4%)	60 (49.6%)	51 (69.9%)	< 0.0001^a^
BASRI-hip score of 4 (*n*, %)	16 (32.7%)	68 (56.2%)	55 (75.3%)	< 0.0001^a^
Preoperative laboratory values				
Elevated level of ESR (*n*, %)	23(46.9%)	75(62.0%)	34(46.6%)	0.058^a^
Elevated level of CRP (*n*, %)	31 (63.3%)	89 (73.6%)	45 (61.6%)	0.168^a^
TT (s)	15.7 ± 0.8	16.2 ± 1.0	16.2 ± 1.0	0.013^b^
APTT (s)	40.6 ± 4.8	41.4 ± 4.7	38.7 ± 4.9	0.001^b^
PT (s)	13.6 ± 0.8	13.5 ± 0.8	13.4 ± 0.9	0.206^b^
PT (%)	93.2 ± 11.3	93.9 ± 11.9	96.1 ± 12.7	0.346^b^
INR	1.06 ± 0.08	1.05 ± 0.08	1.03 ± 0.08	0.219^b^
Fibrinogen level (g/L)	4.04 (2.42 to 7.41)	4.45 (1.90 to 13.10)	4.06 (1.76 to 6.16)	0.014^c^
Platelet level (× 10^9^/L)	270 ± 67	289 ± 77	249 ± 58	0.001^b^
Hb level (g/L)	127 ± 15	131 ± 16	140 ± 16	< 0.0001^b^
Hct (L/L)	0.386 ± 0.041	0.401 ± 0.042	0.423 ± 0.041	< 0.0001^b^
Perioperative data				
Iliopsoas and adductor release (*n*, %)	2 (4.1%)	23 (19.0%)	16 (21.9%)	0.024^a^
Administration of TXA (*n*, %)	40 (81.6%)	77 (63.6%)	42 (57.5%)	< 0.0001^a^
Chemoprophylaxis of thrombosis (*n*, %)	30(61.2%)	97(80.2%)	56(76.7%)	0.033^a^
Operating time (min)	100 (55 to 250)	180 (60 to 335)	210 (70 to 455)	< 0.0001^c^
Autologous transfusion (*n*, %)	3 (6.1%)	30 (24.8%)	33 (45.2%)	< 0.0001^a^
Allogenic transfusion (*n*, %)	37 (75.5%)	119 (98.3%)	73 (100%)	N/A
ASA class				0.672^a^
1	5	6	4	
2	40	105	65	
3	4	10	4	

*ATLS* Advanced Trauma Life Support, *AS* ankylosing spondylitis, *THA* total hip arthroplasty, *BMI* body mass index, *ROM* range of motion, *BASRI-hip* Bath Ankylosing Spondylitis Radiology Hip Index, *ESR* erythrocyte sedimentation rate, *CRP* C-reactive protein, *TT* thromboplastin time, *APTT* activated partial thromboplastin time, *PT* prothrombin time, *PT%* prothrombin activity, *INR* international normalized ratio, *Hb* hemoglobin, *Hct* hematocrit, *TXA* tranexamic acid, *ASA* American Society of Anesthesiologists

^a^Chi-square test

^b^One-way ANOVA

^c^Wilcoxon rank sum test

The ordinal logistic regression results showed that the factors associated with blood loss in AS patients with hip involvement undergoing THA are the male sex (odds ratio [OR] = 3.287; 95% confidence interval [CI], 1.022 to 10.567), bilateral THA (OR = 13.896; 95% CI, 4.950 to 39.011), hip ROM = 0° (OR = 2.513; 95% CI, 1.277 to 4.946), an elevated ESR level (OR = 3.042; 95% CI, 1.320 to 7.014), an elevated preoperative Hb level (OR = 1.043; 95% CI, 1.017 to 1.070), a long operating time (OR = 1.009; 95% CI, 1.003 to 1.016), and the administration of TXA (OR = 0.252; 95% CI, 0.134 to 0.472) (Table 3).

**Table 3:** Ordinal logistic regression of the risk factors for blood loss in AS patients with hip involvement undergoing THA

Parameter	OR (95% CI)	*P* value
Male sex	3.287 (1.022 to 10.567)	**0.046**
BMI	0.960 (0.897 to 1.027)	0.236
Bilateral THA	13.896 (4.950 to 39.011)	**< 0.0001**
Hip ROM = 0°	2.513 (1.277 to 4.946)	**0.008**
BASRI-hip score of 4	0.700 (0.345 to 1.421)	0.324
Elevated ESR level	3.042 (1.320 to 7.014)	**0.009**
Elevated CRP level	0.885 (0.415 to 1.888)	0.752
TT (s)	1.189 (0.880 to 1.606)	0.260
APTT (s)	0.964 (0.901 to 1.032)	0.291
Fibrinogen(g/L)	0.780 (0.562 to 1.081)	0.136
Platelet level( × 10^9^/L)	0.997 (0.992 to 1.002)	0.211
Preoperative Hb level (g/L)	1.043 (1.017 to 1.070)	**0.001**
Iliopsoas and adductor release	1.934 (0.860 to 4.350)	0.111
Administration of TXA	0.252 (0.134 to 0.472)	**< 0.0001**
Chemoprophylaxis of thrombosis	1.814 (0.896 to 3.674)	0.098
Operating time (min)	1.009 (1.003 to 1.016)	**0.004**

*AS* ankylosing spondylitis, *THA* total hip arthroplasty, *OR* odds ratio, *CI* confidence interval, *BMI* body mass index, *ROM* range of motion, *BASRI-hip* Bath Ankylosing Spondylitis Radiology Hip Index, *ESR* erythrocyte sedimentation rate, *CRP* C-reactive protein, *TT* thromboplastin time, *APTT* activated partial thromboplastin time, *Hb* hemoglobin, *TXA* tranexamic acid

## Discussion

THA has been proven to be extremely effective in treating AS patients with advanced hip involvement [3–7], while the large volume of blood loss is a consequence that should be addressed. The blood loss calculated in our study included that of all the bleeding events: intraoperative blood loss, postoperative drainage, and “hidden” blood loss [25]. As mentioned, bony ankylosis at the hip [13] and a higher disease activity [17] increase bleeding during THA in AS patients. Owing to the characteristic differences between AS and other hip diseases requiring THA, we hypothesized that disease activity, the severity of hip disease, and the administration of perioperative medication might be associated with perioperative bleeding in AS patients. Thus, we conducted this relatively comprehensive research on factors related to blood loss during THA in AS patients with hip involvement.

The results of this study indicate that the male sex, bilateral THA, hip ROM = 0°, an elevated ESR level, an elevated preoperative Hb level, and long operating time are risk factors associated with blood loss in AS patients with hip involvement undergoing THA, while the administration of TXA is a protective factor. Being a male is a risk factor for two possible reasons. First, the population of individuals with AS with advanced hip involvement is predominantly male [26]. Second, the average preoperative Hb level is higher in men than in women, as we also identified that an elevated preoperative Hb level is related to bleeding during THA in AS patients. Generally, after bleeding and the blood transfusion process is complete, the Hb levels should remain above the lower limit of the normal range, and patients should not have symptoms of hypovolemic shock. Therefore, according to the principle of Hb balance, a higher preoperative Hb level is expected to result in more blood loss. In addition, autologous transfusion can be a beneficial choice for patients with an elevated preoperative Hb level, as shown in our practice. However, autologous transfusion only is insufficient, and allogenic blood transfusion is also needed. The total allogenic transfusion rate in this study is higher than reported in other studies [13, 17, 18], which is mainly due to the higher proportion of patients undergoing bilateral THA compared with unilateral THA (approximately 2/3). If the allogenic transfusion rate in only patients who underwent unilateral THA is compared between this study and the studies mentioned, our results are comparable.

In our department, the operations were mainly performed by a one-stage operation during one general anesthesia session in patients requiring THA at both hips. Bilateral THA can cause more bleeding than unilateral THA, but several studies have suggested that the total blood loss is significantly lower in patients undergoing a one-stage procedure than in those undergoing a two-stage procedure, and surgeons are probably more concerned about bleeding during a one-stage procedure and therefore take more care to ensure hemostasis [27, 28]. Additionally, the bilateral procedure not only reduces the costs and hospital stay [28] but also yields optimal function in patients with bilateral hip disease [29]. Hence, because the bleeding volume can be more reasonably predicted, appropriate blood management is performed, and hemostasis is ensured during the operation; bilateral THA is still a top option for AS patients who need surgical treatment for both hips.

The reduction in the hip ROM is a characteristic clinical manifestation of severe hip involvement in AS cases. A hip ROM = 0°, which is considered the external manifestation of hip ankylosis, is a marked risk factor associated with blood loss during THA. During the procedure for hip ankylosis, the femoral head cannot be dislocated after exposure, which means that a two-step osteotomy must be performed to ream the acetabulum. A two-step osteotomy increases the operating time, and a long operation at the cancellous bone interface causes increased intraoperative bleeding. To minimize bleeding at the osteotomy surface, we sealed it with bone wax after the first osteotomy and then performed the second osteotomy and acetabulum preparation. Moreover, a long operating time is also a risk factor for excessive bleeding during THA in AS patients. Thus, stanching bleeding at the osteotomy interface and shortening the operating time are important for reducing perioperative bleeding and postoperative transfusion.

ESR and CRP are biomarkers of inflammatory diseases that are commonly examined and can reflect the disease activity to some extent. Similar to Hu et al. [17], we determined that an elevated ESR level is a risk factor for blood loss in AS patients with end-stage hip disease undergoing THA. The frequency of elevated ESR level was higher in group II and did not gradually increase with the grade of blood loss, which could be interpreted as follows: patients in that group were mainly at the transition period from mid- to end-stage AS. This period is the stage during which hip involvement in AS cases is aggravated and accompanied by higher disease activity, which is characterized by continuous narrowing of the joint space until bony ankylosis develops. When the articular cartilage is completely damaged, the disease activity declines [30]. Therefore, it is recommended that ESR level is decreased as much as possible before THA under the care of rheumatologists.

The clinical administration of TXA can effectively reduce perioperative blood loss and the transfusion rate during THA [14, 15, 31–33]. Likewise, we confirmed that the administration of TXA is a protective factor for blood loss during THA in AS patients. Several studies have shown that the administration of TXA does not increase the risk of deep vein thrombosis, pulmonary embolism, or other complications [34, 35], regardless of whether routine chemical thromboprophylaxis is administered after primary THA [36, 37]. In recent years, we also used TXA topically in the surgical fields or intra-articularly before the surgical wounds were closed [38, 39], but TXA was administered by an intravenous injection in this study. In addition, we found that the administration of chemical thromboprophylaxis had no effect on total blood loss during THA, but this factor still needs to be studied further.

Several limitations exist in this study. First, patients were administered TXA and chemical thromboprophylaxis by different surgeons, which can lead to selection bias. TXA has been routinely used in our department in recent years, but it was not used routinely at the beginning of this study period. Second, this is a single-center study, which may lead to admission bias.

In conclusion, our study determined that the male sex, bilateral THA, hip ROM = 0°, an elevated ESR level, an elevated preoperative Hb level, and long operating time are risk factors associated with blood loss in AS patients with hip involvement undergoing THA, while the administration of TXA is a protective factor. Since the preoperative Hb level is related to bleeding, it may be beneficial to prepare for autologous transfusion for patients with a higher preoperative Hb level. These results may help determine the risk of bleeding in perioperative assessments and develop more efficient blood management strategies for THA in AS patients with hip involvement.

## Data Availability

All data generated or analyzed during this study are included in this published article.

## Abbreviations

AS: Ankylosing spondylitis
THA: Total hip arthroplasty
BMI: Body mass index
ROM: Range of motion
BASRI-hip: Bath Ankylosing Spondylitis Radiology Hip Index
ESR: Erythrocyte sedimentation rate
CRP: C-reactive protein
TT: Thromboplastin time
APTT: Activated partial thromboplastin time
PT: Prothrombin time
PT%: Prothrombin activity
INR: International normalized ratio
Hb: Hemoglobin
Hct: Hematocrit
TXA: Tranexamic acid
BV: Blood volume
CBL: Calculated blood loss
ASA: American Society of Anesthesiologists
OR: Odds ratio
CI: Confidence interval
